# Regulatory T Cell-Related Gene Indicators in Pulmonary Hypertension

**DOI:** 10.3389/fphar.2022.908783

**Published:** 2022-05-31

**Authors:** Yan Liu, Jun-Zhuo Shi, Rong Jiang, Shao-Fei Liu, Yang-Yang He, Emiel P. C. van der Vorst, Christian Weber, Yvonne Döring, Yi Yan

**Affiliations:** ^1^ Department of Nuclear Medicine, The First Affiliated Hospital of Zhengzhou University, Zhengzhou, China; ^2^ School of Pharmacy, Henan University, Kaifeng, China; ^3^ College of Traditional Chinese Medicine, Henan University, Kaifeng, China; ^4^ Department of Cardio-Pulmonary Circulation, Shanghai Pulmonary Hospital, Tongji University, Shanghai, China; ^5^ Institute of Physiology, Charité—Universitätsmedizin Berlin, Corporate Member of Freie Universität Berlin, Humboldt-Universität zu Berlin, and Berlin Institute of Health, Berlin, Germany; ^6^ DZHK (German Centre for Cardiovascular Research), Partner Site Berlin, Berlin, Germany; ^7^ Institute for Cardiovascular Prevention, Ludwig-Maximilians-University Munich, Munich, Germany; ^8^ DZHK (German Centre for Cardiovascular Research), Partner Site Munich Heart Alliance, Munich, Germany; ^9^ Interdisciplinary Center for Clinical Research (IZKF), RWTH Aachen University, Aachen, Germany; ^10^ Institute for Molecular Cardiovascular Research (IMCAR), RWTH Aachen University, Aachen, Germany; ^11^ Department of Pathology, Cardiovascular Research Institute Maastricht (CARIM), Maastricht University Medical Centre, Maastricht, Netherlands; ^12^ Department of Biochemistry, Cardiovascular Research Institute Maastricht (CARIM), Maastricht University Medical Centre, Maastricht, Netherlands; ^13^ Munich Cluster for Systems Neurology (SyNergy), Munich, Germany; ^14^ Department of Angiology, Swiss Cardiovascular Center, Inselspital, Bern University Hospital, University of Bern, Bern, Switzerland

**Keywords:** pulmonary hypertension, regulatory T cells, Treg-related genes, transcriptomics, immune, gene indicators

## Abstract

**Objective:** Regulatory T cells (Tregs) are critical immune modulators to maintain immune homeostasis and limit pulmonary hypertension (PH). This study was aimed to identify Treg-related genes (TRGs) in PH.

**Methods:** The gene expression profile from lungs of PH patients was retrieved from the Gene Expression Omnibus (GEO) database. The abundance of Tregs was estimated by the xCell algorithm, the correlation of which with differentially expressed genes (DEGs) was performed. DEGs with a |Pearson correlation coefficient| >0.4 were identified as TRGs. Functional annotation and the protein–protein interaction (PPI) network were analyzed. A gene signature for 25 hub TRGs (TRGscore) was generated by a single sample scoring method to determine its accuracy to distinguish PH from control subjects. TRGs were validated in datasets of transcriptional profiling of PH cohorts and in lung tissues of experimental PH mice.

**Results:** A total of 819 DEGs were identified in lungs of 58 PAH patients compared to that of 25 control subjects of dataset GSE117261. In total, 165 of all these DEGs were correlated with the abundance of Tregs and identified as TRGs, with 90 upregulated genes and 75 downregulated genes compared to that of control subjects. The upregulated TRGs were enriched in negative regulation of multiple pathways, such as cAMP-mediated signaling and I-kappaB kinase/NF-kappaB signaling, and regulated by multiple genes encoding transcriptional factors including HIF1A. Furthermore, 25 hub genes categorized into three clusters out of 165 TRGs were derived, and we identified 27 potential drugs targeting 10 hub TRGs. The TRGscore based on 25 hub TRGs was higher in PH patients and could distinguish PH from control subjects (all AUC >0.7). Among them, 10 genes including *NCF2, MNDA/Ifi211, HCK, FGR, CSF3R, AQP9, S100A8, G6PD/G6pdx, PGD,* and *TXNRD1* were significantly reduced in lungs of severe PH patients of dataset GSE24988 as well as in lungs of hypoxic PH mice compared to corresponding controls.

**Conclusion:** Our finding will shed some light on the Treg-associated therapeutic targets in the progression of PH and emphasize on TRGscore as a novel indicator for PH.

## Introduction

Pulmonary hypertension (PH) is a cardiopulmonary disorder characterized by progressive occlusion of the distal pulmonary arteries and increased pulmonary vascular resistance ultimately leading to right heart failure and even death (Hassoun, 2021). The pathogenesis of PH is driven or initiated by multiple elements such as genetic predisposition (Morrell et al., 2019; Wang et al., 2020), epigenetic alterations (Potus et al., 2020; Yan et al., 2020; Wang Q. et al., 2022), inflammation (Rabinovitch et al., 2014; Liang et al., 2021), altered metabolism (He et al., 2020; He Y.-y. et al., 2021; Xu et al., 2021; Wang S. et al., 2022), and environmental cues such as hypoxia (He Y.-Y. et al., 2021) and growth factors (Yan et al., 2021). It could be categorized into five groups according to the World Health Organization classification (Simonneau et al., 2019). As of today, most of the targeted drugs are developed against group 1 PH (pulmonary arterial hypertension, PAH) including endothelin receptor antagonists, phosphodiesterase type 5 inhibitors, and prostacyclin (Hassoun, 2021). However, the prognosis of PAH is far from satisfactory, with a 5-year survival rate being 57% in REVEAL Registry in the modern management era (Benza et al., 2012). Hence, the discovery of some other targets for PAH would open new avenues for the therapeutic strategies.

The failure to resolve inflammation and altered immune response would lead to the disturbance of delicate balance between immunity and tolerance, which might explain the accumulation of perivascular inflammatory cells and overabundance of cytokines and chemokines. As a consequence, it gives rise to chronic inflammation and autoimmunity underlying the development of PAH. Among all the immune cells, regulatory T cells (Tregs) are the major cell type orchestrating self-tolerance in PAH. It was reported that athymic nude rats (lacking normal T cells) exposed to Sugen 5416 (a vascular endothelial growth factor receptor 2 antagonist that causes pulmonary arteriole injuries) demonstrated an accumulation of B cells and macrophage infiltrates even before developing hemodynamically significant PAH, which was prevented with Tregs reconstitution possibly due to a restoration of bone morphogenetic protein receptor type 2 expression, thus improving endothelial cell survival (Tamosiuniene et al., 2011). In addition, female athymic PAH rats exhibited worse hemodynamic compromise, enhanced macrophage infiltration, and augmented right ventricular fibrosis than males, which could also be mitigated by Treg infusion as well attributable to the raised levels of plasma prostacyclin and other cardiopulmonary vasoprotective proteins (e g., prostacyclin synthase) (Tamosiuniene et al., 2018). Tregs can also control neutrophilic infiltration, possibly relevant to the pathogenesis of PAH (Ring et al., 2021). The lines of evidence demonstrate reduced Tregs function or frequency in PAH patients (Suen and Chiang, 2012; Gaowa et al., 2014; Huertas et al., 2016; Frantz et al., 2018). Since restoring the abundance of Tregs could dampen PAH progression, the discovery of key genes or targets linking with Treg infiltrates is of great importance for Treg accumulation into the inflammatory milieu of PAH. Targeting Tregs for the shape of the immune homeostasis is increasingly considered a promising therapeutic strategy for pulmonary vascular remodeling in PAH (Tian et al., 2021).

Here, we sought to examine Treg-related genes (TRGs) in PAH by virtue of transcriptomic data of lung tissues and Treg infiltrates in datasets of PH patients followed by the validation of those genes in experimental PH mice, which would facilitate our knowledge of Treg-associated molecular mechanisms in PH development. The integration of hub TRGs to form a multigene signature would also allow for the discovery of novel indicator for the distinction of PH from control subjects as well.

## Methods

### Data Sources and Visualization

We retrieved gene expression microarray data of lung tissues from 58 patients with pulmonary arterial hypertension (PAH, group 1 PH) and 25 control subjects with the accession number GSE117261 (Stearman et al., 2019) based on GPL6244 from the Gene Expression Omnibus (GEO) database. A volcano plot was generated for the distribution of distinguishing genes between PAH patients and control subjects. To validate the hub genes, two independent datasets were downloaded based on GPL6244. One dataset [GSE24988 (Mura et al., 2012)] included gene profiling of lungs from 17 severe PH patients and 22 controls, and the other [GSE113439 (Mura et al., 2019)] profiled the gene expression of lungs from 15 PH patients and 11 controls.

### Evaluation of Treg Infiltration

A web tool, xCell (Aran et al., 2017) (https://xcell.ucsf.edu), was utilized to acquire the abundance of Tregs, which is also able to depict the enrichment of 64 immune and stroma cell types for the better understanding of cellular heterogeneity landscape of human tissue expression profiles.

### Identification of Treg-Related Genes and the Functional Enrichment

Differentially expressed genes in lungs between PH and control subjects were identified using the limma package in R (v3.6.3.). The genes with a fold change (FC) > 1.5 or <0.67 between PH and controls and *p* < 0.05 were considered to be differentially expressed genes (DEGs). The relationship of all DEGs with the abundance of Tregs was further investigated by Pearson correlation analysis. DEGs with a |Pearson correlation coefficient| > 0.4 were regarded as TRGs. The expression of TRGs in the individual sample was plotted in a heatmap. Correlation of Treg infiltrates with a specific gene was plotted in scatterplot in R. Pathway enrichment of the indicated genes were obtained in the Database for Annotation, Visualization, and Integrated Discovery (Huang da et al., 2009b; a) (DAVID, v6.8, https://david.ncifcrf.gov/tools.jsp). The genes encoding transcriptional factors in regulation of TRGs were identified by TRRUST from the functional annotation tool Metascape (Zhou et al., 2019) (https://metascape.org/gp/index.html#/main/step1).

### Protein–Protein Interaction Network and Identification of Hub Genes

PPI network was constructed by STRING (Szklarczyk et al., 2019) (v11.0, https://string-db.org), a biological web resource, for systemic screening of human protein interactions. The network was then visualized in Cytoscape (Shannon et al., 2003) (v.3.8.2). Hub TRGs of the network were identified by Molecular Complex Detection (MCODE) plugin in Cytoscape (nodes ≥ 5 and node score cutoff ≥ 5) as previously described (Yan et al., 2021).

### Construction of a Hub TRG Signature

For dimensional reduction of transcriptomic data, the function “singscore” (Foroutan et al., 2018) in the R package was used to score a gene expression dataset against a gene signature at a single-sample level. This method provides stable scores, which are particularly useful for small sample size and also less likely to be affected by gene sizes. In our study, the score against hub TRGs were termed as TRGscore and calculated for lungs of GSE117261, GSE24988, and GSE113439 datasets. Receiver operation characteristics (ROC) curve analysis was performed to assess the diagnostic accuracy of TRGscore for PH using “pROC” package in R.

### The Drug–Gene Interaction Analysis

The drug–gene interaction analysis was performed with the Drug–Gene Interaction Database (DGIdb, version 4.2.0; www.dgidb.org), an online platform that consolidates disparate data sources describing drug–gene interactions and gene druggability (Freshour et al., 2021). A list of hub TRGs was used as input genes. Drugs with Query score > 4 and Interaction Score >1 were considered to be potential small molecular compounds that can modulate activity or expression of the user-defined gene list in the database.

### Animal Experiment

The experiment was carried out in accordance with the Guideline for Care and Use of Laboratory Animals published by the US National Institutes of Health and the Guidelines for the ethical review of laboratory animal welfare People’s Republic of China National Standard GB/T 35892-2018 and approved by the Ethics and Animal Care and Use Committee of Henan University (Henan, China) (MacArthur Clark and Sun, 2020). A total of 16 male 8-week-old C57BL/6 mice were provided by Vital River Laboratories Co., Ltd. (Beijing, China) and housed under specific pathogen-free conditions (12 h light/12 h dark photoperiod, 25 ± 2°C, and 50 ± 5% relative humidity). The mice were randomly kept either in the hypoxia chamber (10% oxygen) or in room air for 4 weeks (n = 8/condition). The feeding, water, and cages for mice were changed twice a week.

### Hemodynamics and Right Ventricular Hypertrophy Assessment

To examine the establishment of PH, all mice were weighed and anesthetized with pentobarbital (30 mg/kg) at day 28. Right heat catheterization was performed using a Millar catheter to measure right ventricular systolic pressure (RVSP). All hemodynamic data were analyzed using the PowerLab data acquisition system (Power Lab 8/30; AD Instruments, Sydney, Australia). The mice were euthanized, and the heart was harvested after catheterization. Next, the right ventricular free wall was removed from the left ventricle (LV) and septum (S). Right ventricular hypertrophy was assessed by the ratio of the weight of the right ventricle (RV) to the weight of the LV and septum [RV/(LV + S)].

### Pulmonary Arterial Wall Thickness Measurement

Mouse lungs were isolated and perfused with cold phosphate-buffered saline (pH 7.4) to remove blood and then fixed in a 10% formalin overnight. The fixed lung tissues were dehydrated and further processed for hematoxylin and eosin (H&E) staining. The pulmonary arterial wall thickness was calculated as (external vessel area-internal vessel area)/(external vessel area), as previously described (Yan et al., 2020).

### cDNA Synthesis and Real Time Polymerase Chain Reaction

The total RNA was isolated from frozen mouse lung tissues with TRIzol (Invitrogen), followed by reverse transcription (cDNA synthesis) using the SuperScript®III First-Strand Synthesis System (Invitrogen) according to the manufacturer’s instructions. RT-PCR was then performed with the FastStart Universal SYBR Green Master (ROX) (Roche) kit in the ABI 7500 real-time detection system. The threshold cycle (Ct) values of the target genes were normalized to that of the housekeeping gene (*Gapdh*). All data were analyzed by the 2^−ΔΔCt^ method, as previously described (Yan et al., 2020). The average of the relative mRNA expression from control samples was set at 1. Primers were purchased from Tsingke Biotechnology Co., Ltd. (Beijing, China), and the sequences are listed in Table 1.

**TABLE 1:** Primers for the hub TRGs to be validated by RT-PCR in mouse lungs of hypoxia-induced PH.

Gene_mouse	Forward primer	Reverse primer
*Ncf2*	ATT​AGT​CAT​CAA​CAC​GAG​GAG​T	TAG​ATG​TGC​AGC​CAC​TAC​ACT
*Ifi211*	GTG​CCC​AAC​TGT​ATT​ACC​AGA	TTG​ATG​TTG​TGC​CAT​TGT​CCA
*Itgam*	AGC​CCC​AAG​AAA​GTA​GCA​A	TCA​TCA​AAG​AAG​GCA​CGG​AT
*Hck*	CCA​TCT​TTA​TTG​TCA​CGG​AGT	TTC​CAT​CAG​CAG​GAT​ACC​AA
*Fgr*	CAT​TAG​GCT​CCG​ACA​TAC​CG	ACA​CAC​ATA​CAC​GTC​CCT​C
*Cxcr1*	ACT​GGA​GAT​TAT​TTC​ATC​CCC​T	CAT​CAC​CAG​CGA​GTT​TCC​C
*Csf3r*	TGG​CCC​AAG​ACC​TTA​ACA​CC	AGC​CAC​TGT​AAT​TCT​GTA​GAG​C
*Aqp9*	CTT​TGA​TTT​CCA​TAC​GCC​AT	ATT​TAT​ATG​CCC​ATA​GGT​TGC
*S100a9*	CAT​CTG​TGA​CTC​TTT​AGC​CTT	TCA​ACT​TTG​CCA​TCA​GCA​TC
*S100a8*	TGC​CCT​CTA​CAA​GAA​TGA​CT	ACT​CCT​CGA​AGT​TAA​TTG​CAT
*Eno1*	AGA​CAA​TGA​TAA​GAC​CCG​CTT​C	ATT​TAT​TCT​CTG​TGC​CGT​CCA
*Gsr*	TTC​CAG​CTA​TAC​ACA​CGC​ACA	CCA​GCA​ATA​TTC​ATC​CAT​CAG​GT
*G6pdx*	CCT​GGC​ATG​TTC​TTT​AAC​CC	TTC​TCG​ATC​AAT​CTT​GTG​CAG
*Pgd*	GTT​CAC​TTT​TAA​CTC​CGG​CTT	TGC​CAG​TAA​GAA​ATA​CCC​GAC​A
*Txnrd1*	ATT​TAT​CCC​CAG​AAA​GAC​GAA	GCA​GTT​CAA​GTA​GAT​TAG​CCA
*Tkt*	ATG​TCC​ACC​GTC​TTT​TAC​CCA​A	GCC​TCA​TGC​AGA​GTT​ACA​CCA
*Taldo1*	TCA​CCA​TCT​CCC​CGA​AGC​TC	ATG​ATC​CAT​CCC​TTC​TCC​GAT
*Pde8a*	TAG​AGA​ACC​CCA​ACG​TCA​TGG​C	TGG​CAT​CGA​GCA​AAT​CAC​CT
*Pde7b*	AAA​CAT​TGT​CTT​TAG​CCA​GT	TTC​CAA​GAG​AGC​CAT​TAG​CAG
*Pde4d*	TGT​CCT​TGT​AGT​CAC​CCG​AAC	TAG​GCA​TTA​AAC​CAA​GCA​ACA​GG
*Pde2a*	CGG​CTG​CAA​TAT​CTT​TGA​CCA​C	TTG​TTT​CGG​TCA​TAA​CCC​ACT
*Pde1a*	ATA​AAA​TGT​TTG​GCT​CAG​TCC	CCT​AAT​GAT​AAC​TCG​TGG​GA
*Gapdh*	CTC​ACG​GCA​AAT​TCA​ACG​G	AGT​TGT​CAT​ATT​TCT​CGT​GGT

### Statistics

The data were presented as the mean ± standard error of the mean (SEM). Statistical differences between two groups were evaluated with two-tailed unpaired Student’s *t*-test if the samples were normally distributed. Otherwise, the Mann–Whitney test was used to detect the difference (GraphPad Prism 8). *p* < 0.05 was considered to be statistically significant.

## Results

### Identification of TRGs in Lungs of PAH Patients

**FIGURE 1:**
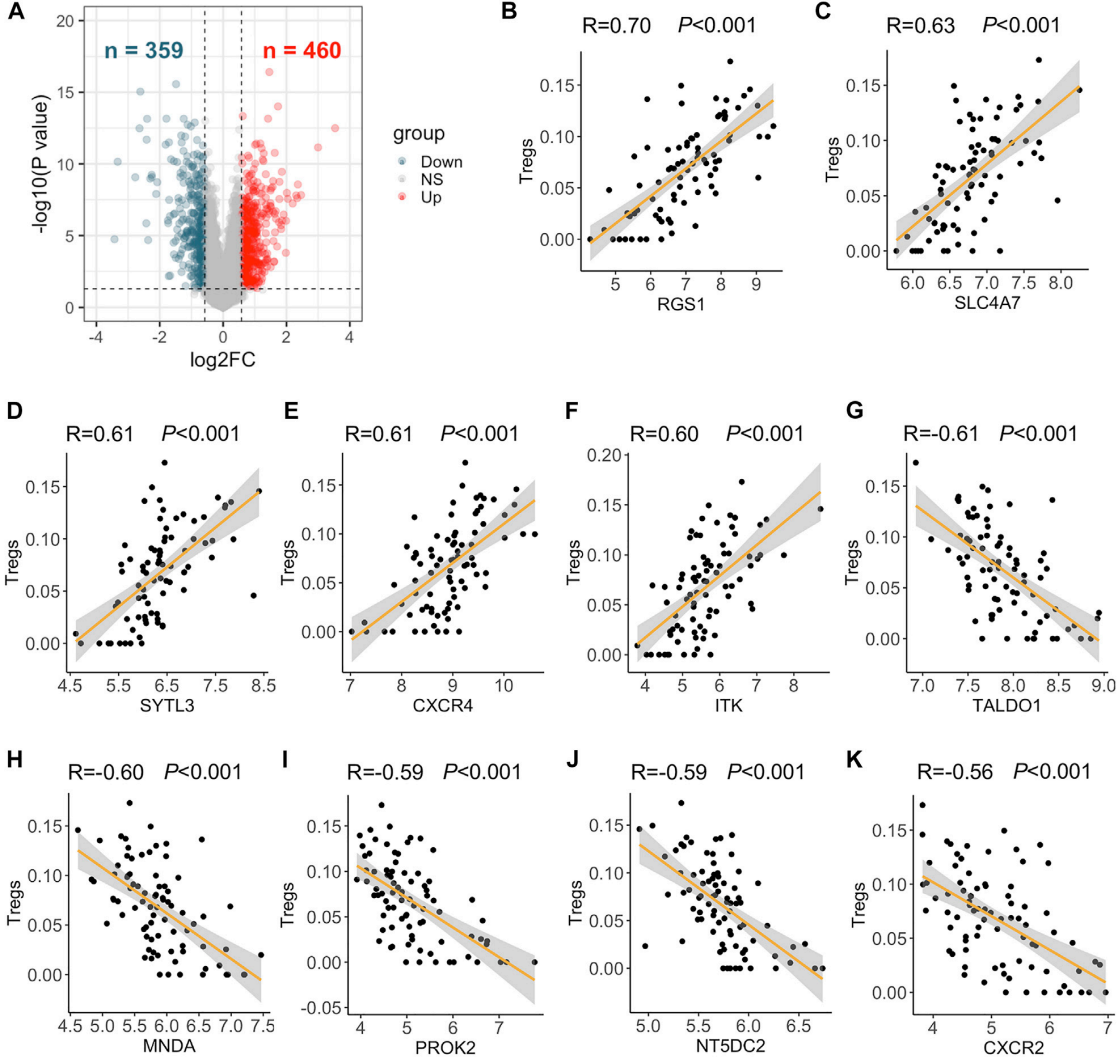
Differentially expressed genes (DEGs) and top five DEGs positively and negatively correlated with the abundance of Tregs. **(A)** DEGs between 58 PAH patients and 25 control subjects of the GSE117261 dataset were visualized in volcano plots (fold change >1.5 or <0.67 and *p* < 0.05). Red dot represents upregulated DEGs, and dark blue dot represents downregulated DEGs in PAH patients compared to control subjects. **(B–F)** *RGS1* **(B)**; *SLC4A7* **(C)**; *SYTL3* **(D)**; *CXCR4* **(E)**; and *ITK* **(F)** were DEGs most positively correlated with the abundance of Tregs as analyzed by Pearson correlation analysis. **(G–K)** *TALDO1* **(G)**; *MNDA* **(H)**; *PROK2* **(I)**; *NT5DC2* **(J)**; and *CXCR2* **(K)** were DEGs most negatively correlated with the abundance of Tregs as analyzed by Pearson correlation analysis.

First, a total of 819 DEGs were identified in lungs of 58 PAH patients compared to those of 25 control subjects of the GSE117261 dataset, with 460 upregulated and 359 downregulated genes (Figure 1A). The Treg composition of each lung sample of this dataset was then explored by the web tool xCell. It turned out that 165 DEGs were correlated with the abundance of Tregs (|correlation coefficient| > 0.4) and were identified as TRGs. *RGS1* (*R* = 0.70), *SLC4A7* (*R* = 0.63), *SYTL3* (*R* = 0.61), *CXCR4* (*R* = 0.61), and *ITK* (*R* = 0.6) were considered the top 5 DEGs positively correlated with Treg abundance (all *p* < 0.001) (Figures 1B–F). *TALDO1* (*R* = −0.61), *MNDA* (*R* = −0.60), *PROK2* (*R* = −0.59), *NT5DC2* (*R* = −0.59), and *CXCR2* (*R* = −0.56) were the top 5 genes in negative correlation with Treg abundance (all *p* < 0.001) (Figures 1G–K). The expression of 90 upregulated and 75 downregulated TRGs were displayed in each sample of the GSE117261 dataset including 25 control subjects and 58 PAH patients (Supplementary Figure S1).

### Functional Annotation of the Altered TRGs

**FIGURE 2:**
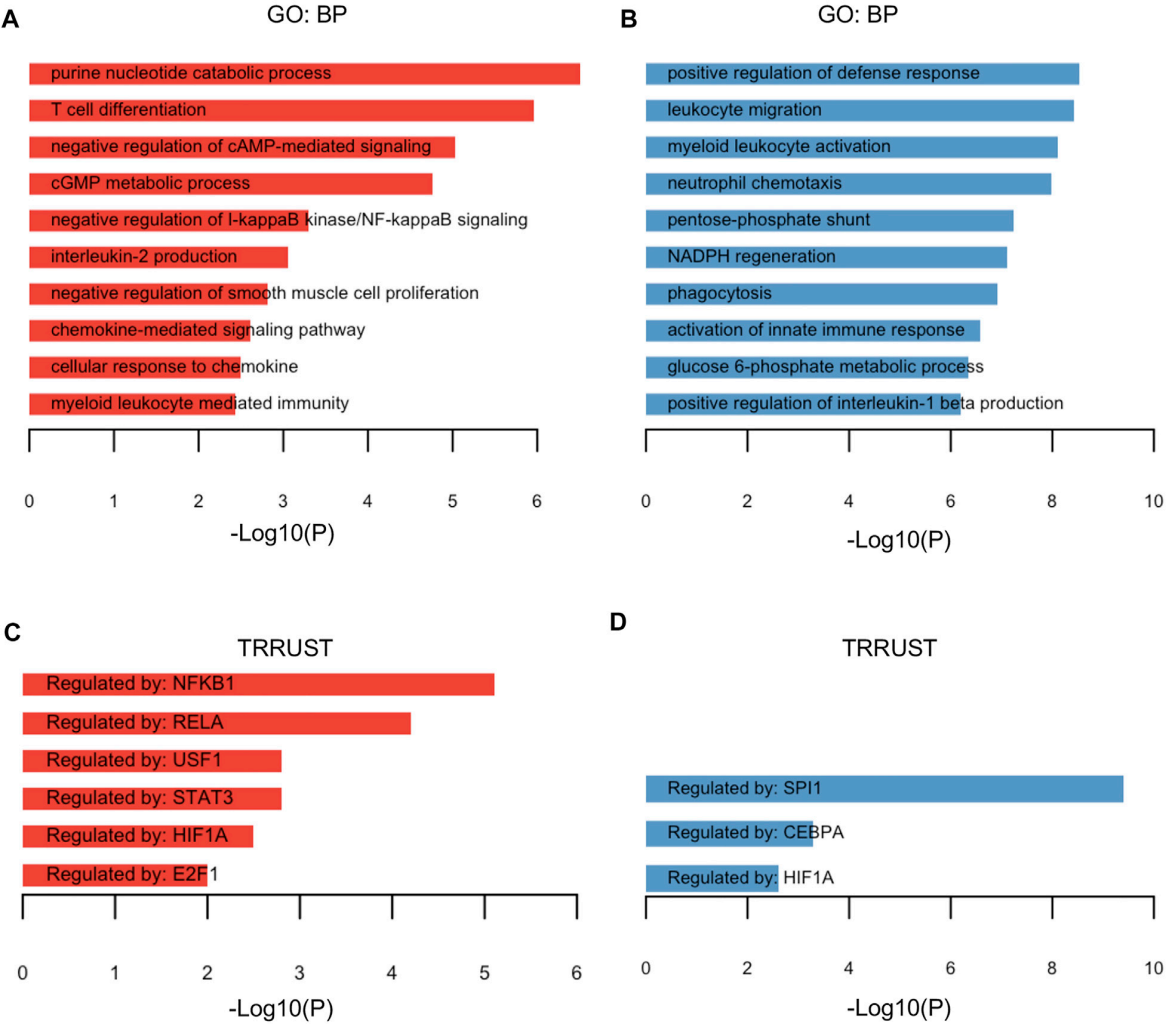
Functional annotation of TRGs. **(A,B)** Gene Ontology—biological pathways for 90 upregulated TRGs **(A)** and 75 downregulated TRGs **(B)** of the aforementioned dataset were identified by the functional annotation web tool DAVID and visualized in the barplot. **(C,D)** Genes encoding transcriptional factors in regulation of the gene profiling of upregulated TRGs **(C)** and downregulated TRGs **(D)** were explored and identified by the TRRUST category from the functional annotation tool Metascape.

To explore pathway enrichment of the upregulated/downregulated TRGs, the DAVID (v6.8) web tool was exploited to unveil the altered Gene Ontology—biological processes. The upregulated TRGs were mainly enriched in pathways such as purine nucleotide catabolic process, negative regulation of cAMP-mediated signaling, negative regulation of I-kappaB kinase/NF-kappaB signaling, and negative regulation of smooth muscle cell proliferation (Figure 2A), while the downregulated TRGs were involved in pathways including positive regulation of defense response, leukocyte migration, myeloid leukocyte activation, neutrophil chemotaxis, and glucose 6-phosphate metabolic process (Figure 2B). Next, the potential transcriptional factors regulating the specific gene profiling were investigated. In line with the enrichment in negative regulation of I-kappaB (IκB) kinase /NF-kappaB (NF-κB) signaling, NFKB1 encoding transcriptional factor NF-κB was identified as one of the genes by the TRRUST category from the functional annotation tool Metascape. Five other genes encoding transcriptional factors (RELA, USF1, STAT3, HIF1A, and E2F1) were also demonstrated to coordinate the upregulation of TRGs (Figure 2C). The downregulated TRGs were regulated by SPI1, CEBPA, and HIF1A (Figure 2D). The upregulated and downregulated TRGs orchestrated by HIF1A underscores the possible linking between Tregs and hypoxia response.

### Identification of Hub TRGs

**FIGURE 3:**
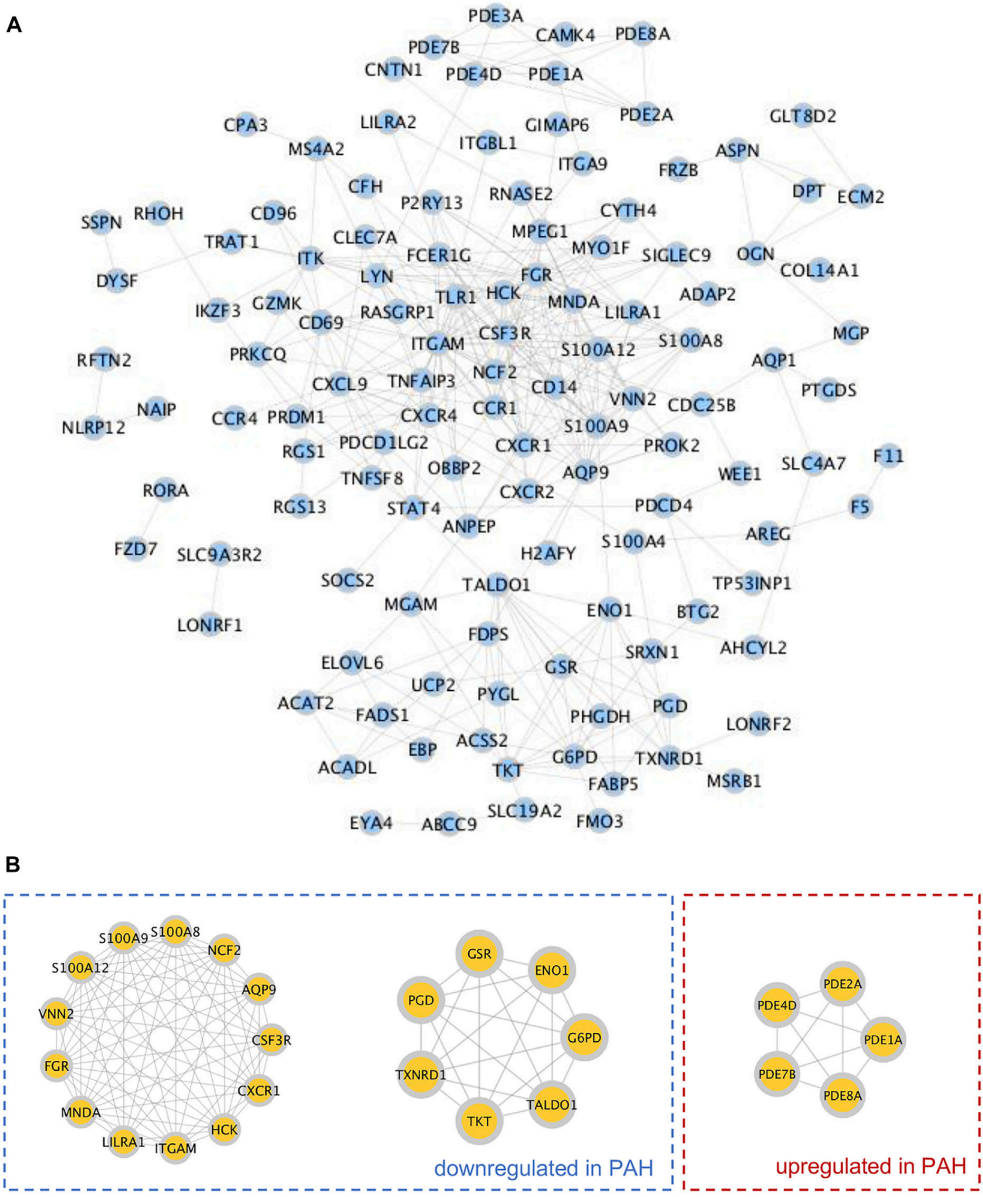
Construction of the protein–protein interaction (PPI) network and identification of hub TRGs. **(A)** PPI network was constructed by the STRING database, and each blue filled circle represents a TRG. **(B)** Hub genes were screened out by the MCODE plugin in Cytoscape and categorized into three clusters. Hub TRGs in the left (first) and middle (second) clusters were downregulated in lungs of PAH patients compared to those of controls of the GSE117261 dataset. The hub TRGs in the right (third) cluster were upregulated in lungs of PAH patients compared to those of controls in the same dataset.

The STRING database was then utilized to construct the PPI network of 165 TRGs, which contained 155 nodes and 368 edges (Figure 3A). According to the MCODE plugin analysis in Cytoscape, 25 TRGs were screened out as hub TRGs, which were categorized as 3 clusters. In total, 13 TRGs with 73 edges contained in the first (left) cluster and 7 TRGs with 21 edges in the second (middle) cluster were lower in lungs of 58 PH patients than those of 25 control subjects. Five hub TRGs encoding some phosphodiesterase (PDE) family members were categorized into the third (right) cluster with 10 edges and higher in PH patients than those in the controls (Figure 3B).

### Discovery of Potential Drugs in Modulation of Hub TRGs

Next, we explored the potential drugs capable of regulation of hub TRGs in the Drug–Gene Interaction Database. A total of 202 drugs targeting 18 hub genes were identified (data not shown). To further narrow down the high-efficiency drugs, serving as antagonist or agonist for the hub TRGs, we selected the targeted drugs with Query Score >4 and Interaction Score >1. Finally, 27 potential existing drugs were figured out to target 10 hub TRGs, including *CSF3R* (4 drugs), *CXCR1* (2 drugs), *G6PD* (9 drugs), *GSR* (3 drugs), *PDE2A* (1 drug), *PDE4D* (4 drugs), *PDE7B* (1 drug), *S100A12* (1 drug), *S100A9* (2 drugs), and *TXNRD1* (1 drug) (Supplementary Table S1). Rasburicase ranked the top of all the drugs relevant to *G6PD* according to Query Score and Interaction Score. Pegfilgrastim was the drug with the highest Query Score and Interaction Score among all the 27 drugs.

### Validation of Hub TRGs in Independent PH Cohorts

**FIGURE 4:**
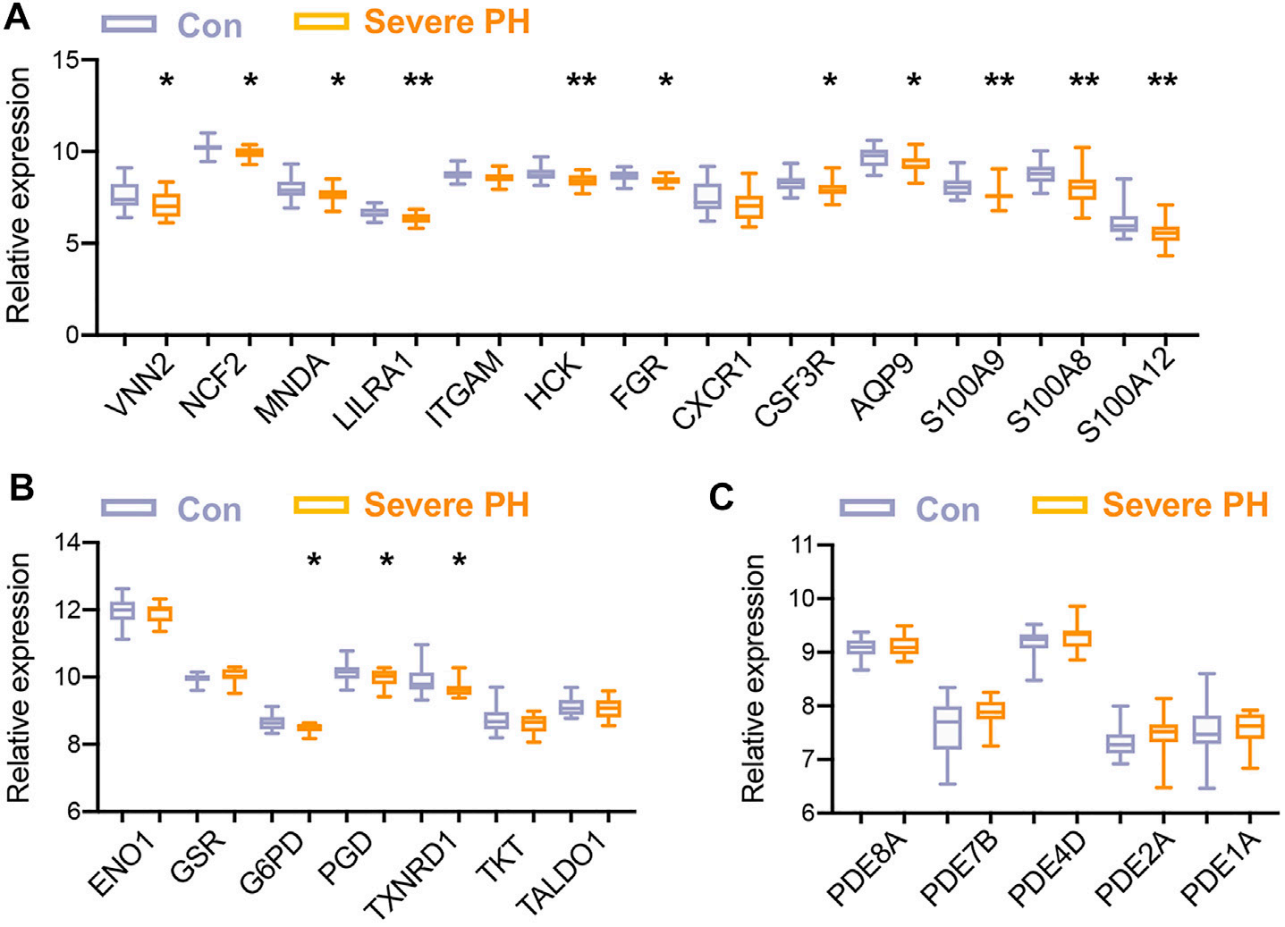
Validation of hub TRGs in a dataset of an independent PH cohort. **(A–C)** Gene expression of hub TRGs in the first (left) cluster **(A)**, hub TRGs in the second (middle) cluster **(B)**, and hub TRGs in the third (right) cluster **(C)** of Figure 3B were examined in lungs of 17 severe PH patients and 22 controls of the GSE24988 dataset. Data represent mean ± SEM. *p < 0.05; **p < 0.01 compared to control subjects, as analyzed by the unpaired *t*-test or Mann–Whitney test as appropriate.

For the validation of the hub TRGs, two independent PH datasets on the same platform (GPL6244) were retrieved. One dataset GSE24988 consisted of gene expression of lungs from 17 severe PH patients and 22 controls. The other dataset GSE113439 contained the gene profiling of lungs from 15 PH patients and 11 controls. As shown in Figures 4A,B, 11 out of 13 (85%) genes in the first cluster and 3 out of 7 (43%) genes in the second cluster were significantly less expressed in severe PH patients than the control subjects of dataset GSE24988. Four hub TRGs in the third cluster tend to be higher in lungs of severe PH patients without reaching any statistical significance (Figure 4C). Next, we explored the hub TRGs in the GSE113439 dataset. It was shown that *MNDA*, *AQP9*, *ENO1*, *PGD*, *TXNRD1*, *PDE8A*, *PDE4D*, and *PDE1A* were higher and *CSF3R*, *G6PD,* and *TALD O 1* were lower in lungs of PH patients (Supplementary Figure S2).

### Clinical Implication of the Hub Gene Signature

**FIGURE 5:**
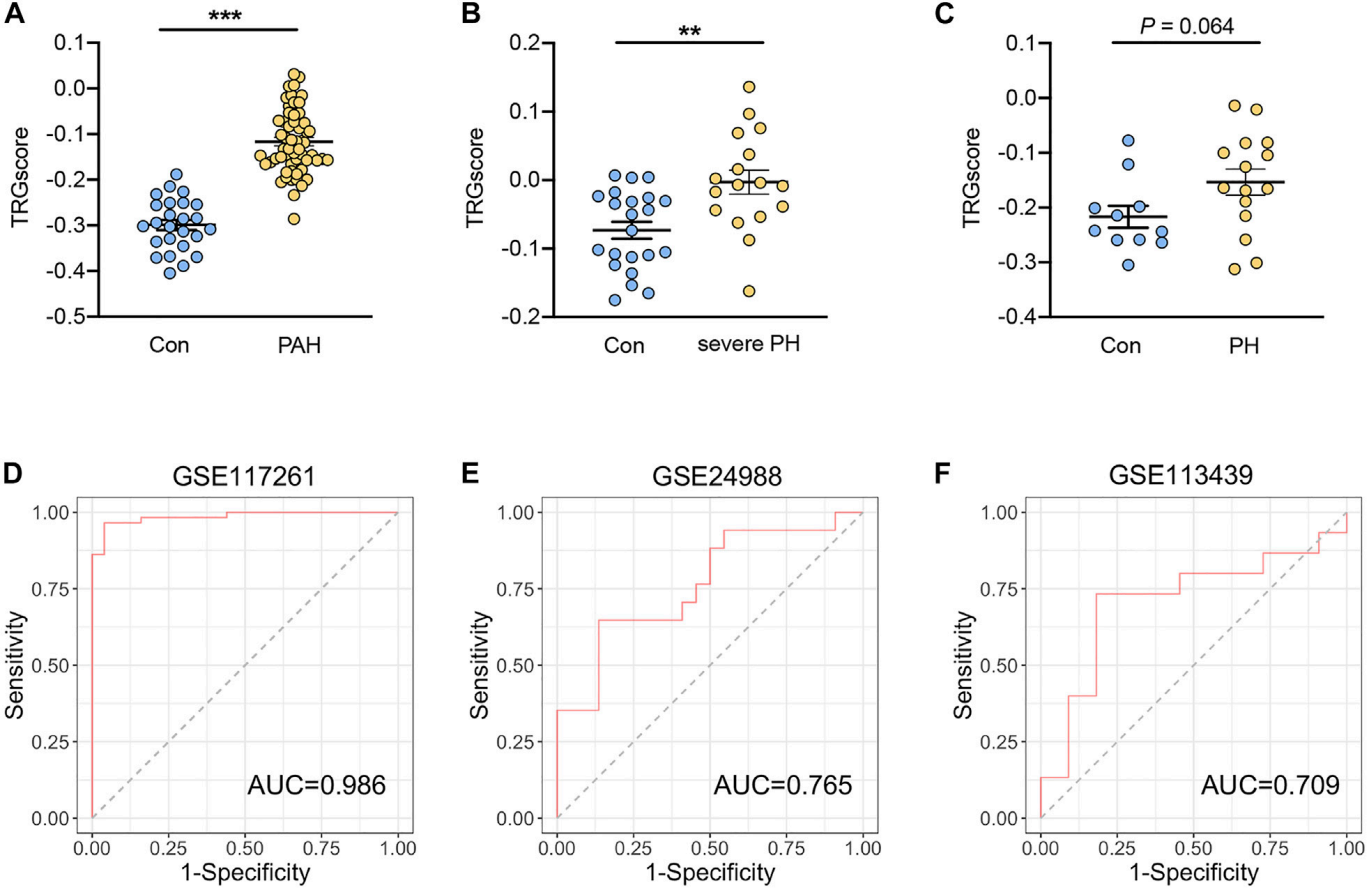
Diagnostic performance of TRGscore for the patients with PH. **(A–C)** TRGscore between patients with PAH (group 1 PH) from the GSE117261 dataset **(A)**, severe PH from the GSE24988 dataset **(B)**, and PH from the GSE113439 dataset **(C)** were compared with the corresponding controls, respectively. **(D–F)** Receiver operation characteristic curves of TRGscore to distinguish PH from controls in GSE117261 **(D)**, GSE24988 **(E)**, and GSE113439 datasets **(F)**, respectively. Data represent mean ± SEM. **p < 0.01; ***p < 0.001 compared to control subjects, as analyzed by the unpaired *t-*test.

To investigate whether the hub TRGs are beneficial for the identification of patients with PH, the 25 hub genes were aggregated to calculate TRGscore according to “singscore” algorithm. PAH (group 1 PH) patients in the GSE117261 dataset and severe PH patients in the GSE24988 dataset exhibited a higher TRGscore than corresponding controls in each dataset (both *p* < 0.05) (Figures 5A,B). In addition, PH patients tend to have a higher TRGscore in the GSE113439 dataset (*p* = 0.064) (Figure 5C). To examine the accuracy to distinguish PH from control subjects, ROC analysis was performed. The result indicated that the area under the curve (AUC) of TRGscore to discriminate PAH patients was 0.986 in the GSE117261 dataset (Figure 5D). Similarly, TRGscore also had a high accuracy for PH diagnosis in the GSE24988 dataset (AUC = 0.765) (Figure 5E) and that of GSE113439 (AUC = 0.709) (Figure 5F).

### Validation of Hub TRGs in Hypoxia-Induced PH Mice

**FIGURE 6:**
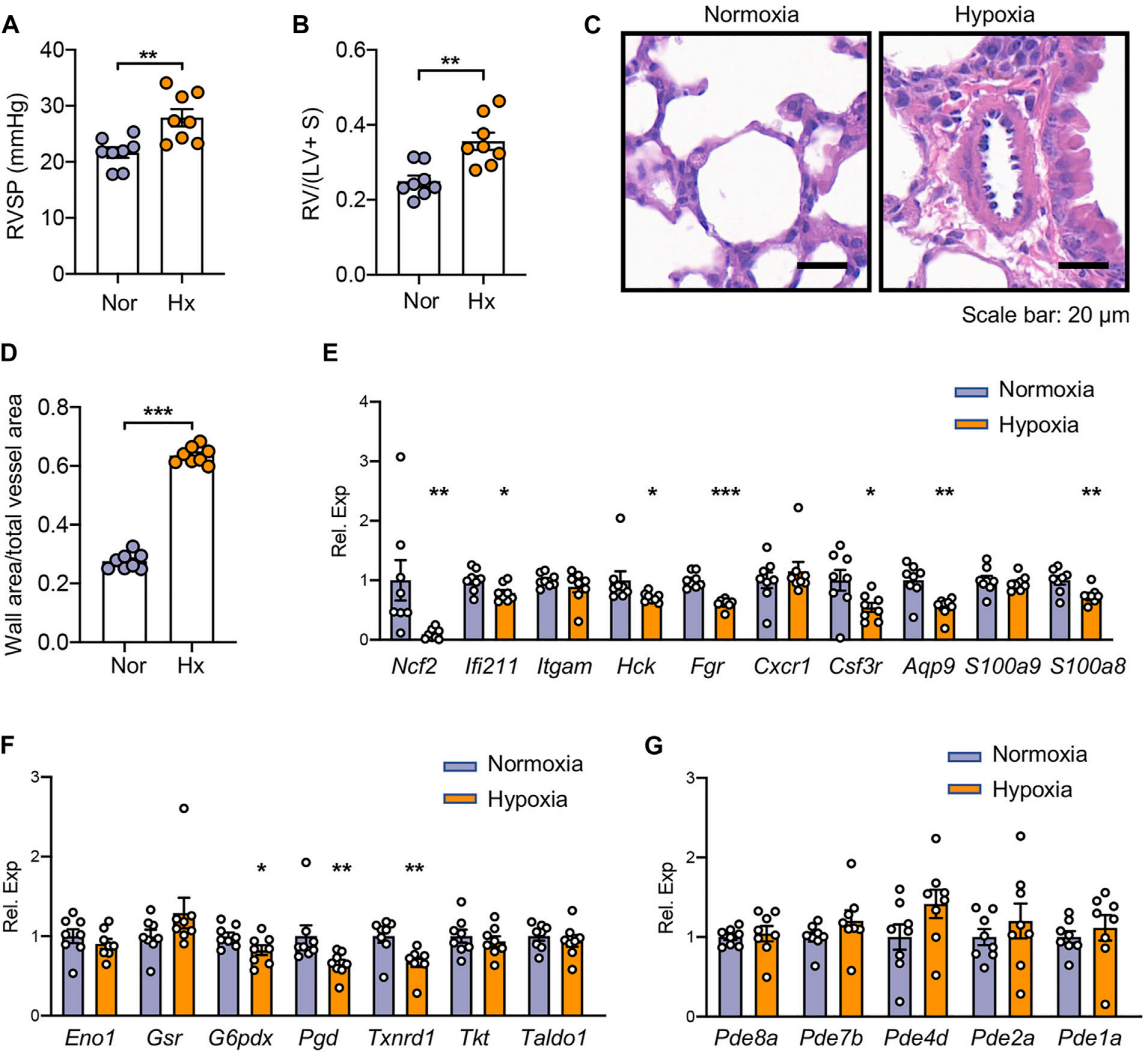
Validation of hub TRGs in mouse lung tissues of hypoxic PH. **(A–B)** Right ventricular systolic pressure (RVSP) **(A)** and right ventricular hypertrophy **(B)** were assessed in hypoxia (Hx)-induced PH mice or mice under normoxia (Nor) at day 28 (n = 8/group). **(C–D)** Representative images of H&E staining **(C)** and quantification of vascular medial thickness **(D)** of lung tissues from mice under hypoxic or normoxic conditions at day 28 (n = 8/group). **(E–G)** Gene expression of hub TRGs in the first (left) cluster **(E)**, in the second (middle) cluster **(F)**, and in the third (right) cluster **(G)** of Figure 3B were examined in lungs of hypoxic PH mice and control mice at day 28 (n = 8/group). Data represent mean ± SEM. *p < 0.05; **p < 0.01; ***p < 0.001 compared to mice under normoxia, as analyzed by the unpaired *t*-test or Mann–Whitney test as appropriate.

To validate the hub TRGs in experimental PH, the mice were exposed to hypoxia for 4 weeks to develop PH. As can be seen from Figure 6A, hypoxic PH mice displayed higher RVSP than the control mice under normoxia (27.89 ± 0.94 mmHg vs. 21.66 ± 1.53 mmHg; *p* < 0.01). Right ventricular hypertrophy was also documented in hypoxic PH mice with a 1.4-fold increase of the ratio of RV mass to the total mass of the left ventricle and septum compared to control mice (*p* < 0.01) (Figure 6B). In addition, the hypoxia exposure elicited the increase of media wall thickness (media hyperplasia) of pulmonary arteries in PH mice relative to control mice in room air (0.64 ± 0.01 vs. 0.28 ± 0.01; *p* < 0.001) (Figures 6C,D). A total of 10 out of 13 genes in the first (left) cluster in Figure 3B have homologs in mice, and seven out of these genes including *Ncf2*, *Ifi211*, *Hck*, *Fgr*, *Csf3r*, *Aqp9*, and *S100a8* were significantly reduced in the lungs of hypoxic PH mice compared to those of mice under normoxia (Figure 6E). As shown in Figure 6F, lower expression of *G6pdx*, *Pgd*, and *Txnrd1* was also displayed in lungs of hypoxic PH mice, which was consistent with the alternation in lungs of severe PH patients. *Pde4d* tends to be higher in lungs of hypoxic PH mice although it did not reach any statistical significance (Figure 6G).

## Discussion

In our study, a total of 165 TRGs (90 genes upregulated and 75 downregulated) were identified in the transcriptional profiles of lungs of PAH patients. The altered TRGs were mainly involved in pathways pertaining to metabolic alteration or inflammatory responses. Our findings showed that both upregulated and downregulated TRGs orchestrated by HIF1A pinpointed a proclivity to PAH possibly due to immunopathology under hypoxia exposure. In the PPI network, 25 hub genes were figured out and categorized into three clusters. Intriguingly, the 25-hub gene signature was capable of discriminating PH patients from control subjects. In addition, we found a list of 27 potential drugs that might modulate the activity or expression of 10 candidate hub TRGs. We also demonstrated that 10 genes including *NCF2*, *MNDA/Ifi211*, *HCK*, *FGR*, *CSF3R*, *AQP9*, *S100A8*, *G6PD/G6pdx*, *PGD*, and *TXNRD1* were significantly reduced in lungs of severe PH patients of the GSE24988 dataset as well as in lungs of hypoxic PH mice compared to the corresponding controls.

The upregulated TRGs were found to be not only associated with Treg infiltrates but also involved in pathways linking to suppressive function of Tregs such as negative regulation of IκB kinase/NF-κB signaling and negative regulation of cAMP-mediated signaling. Although NF-κB is a pro-inflammatory transcription factor that induces Th17-related cytokine genes and the retinoid-related orphan receptor-g that specifies Th17 differentiation, two NF-κB proteins (c-Rel and p65) drive the Treg polarization by promoting the formation of a Foxp3-specific enhanceosome (Ruan and Chen, 2012). This study pinpoints the importance of NF-κB signaling for the development of both anti-inflammatory Tregs and pro-inflammatory Th17 cells. Since the reduced IκB kinase/NF-κB signaling might lead to compromised Tregs and Th17 biology, the balance between pro-inflammatory Th17 and immunosuppressive Tregs in the milieu of the pulmonary vasculature would determine the progression of remodeling. cAMP is considered to be a potent inhibitor of proliferation and pro-inflammatory interleukin 2 synthesis in T cells (Bodor et al., 2001). It was reported that the suppressive activity of naturally occurring Tregs was abrogated by a cAMP antagonist as well as by a gap junction inhibitor, which impeded the cell contact transfer of cAMP to responder T cells (Bopp et al., 2007). This supports the notion that cAMP is crucial for naturally occurring Treg-mediated suppression. The involvement of the cGMP metabolic process was also able to stimulate the hydrolytic activity of phosphodiesterase 2A (PDE2A) in conventional T cells, which was found to be higher in PAH of the GSE117261 dataset, to further reduce cAMP levels (Kurelic et al., 2021). The enrichment in negative regulation of cAMP-mediated signaling restrains immunomodulatory properties in Tregs and favors inflammatory response, possibly relevant to the pathogenesis of PAH.

Hypoxia is a critical stress impacting the biological activity of mammals and would lead to multiple diseases after maladaptation (Li F. et al., 2021; Li M. et al., 2021). In our study, the altered TRGs were demonstrated to be regulated by HIF1A-encoding hypoxia-inducible factor-1α (HIF-1α), which is a critical transcriptional factor activated under hypoxic conditions to determine whether pyruvate is diverted to lactate for glycolysis or to acetyl-CoA to enter the tricarboxylic acid cycle for oxidative phosphorylation. HIF-1α enhances the conversion of pyruvate to lactate and favors a glycolysis-shift *via* the augmentation of glycolytic-related genes, lactate dehydrogenase enzyme activity, and pyruvate dehydrogenase kinase 1, which subsequently inhibits pyruvate dehydrogenase and reduces the transformation of pyruvate to acetyl-CoA (Kim et al., 2006). In contrast to effector T cells, which mainly use glycolysis following stimulation, Tregs have a quite different metabolic reprogramming in favor of oxidative phosphorylation and fatty acid oxidation for their proliferation and functionality (Kempkes et al., 2019). In support of the notion, it was reported that the inhibition of HIF-1α-dependent glycolytic pathways could steer T cells toward Treg differentiation (Shi et al., 2011). Nonetheless, the exact role of HIF-1α in Treg differentiation is controversial. HIF-1α was also reported to promote FOXP3 expression (a very critical transcriptional factor for Treg differentiation) and to be indispensable for Treg development and function (Clambey et al., 2012). Lines of evidence show that HIF-1α was increased in human and experiment PAH (Bonnet et al., 2006; Lei et al., 2016). Cumulatively, this might indicate that HIF-1α promotes pulmonary vascular remodeling *via* the orchestration of immunopathology relevant to cell biology in Tregs.

Rasburicase topped the list of the potential drugs relevant to *G6PD*. It is a recombinant form of urate oxidase that oxidizes uric acid to allantoin, leading to a reduction of serum uric acid levels. A previous study reported that rasburicase was applied to treatment of acute hyperuricemia and renal dysfunction (Ronco et al., 2005). As the uric acid metabolism was disturbed in remodeled pulmonary vasculature in both experimental and human PAH, and uric acid levels in idiopathic PAH were associated with a compromised clinical and hemodynamic profile (Savale et al., 2021), it could be postulated that rasburicase might be suitable for PAH patients especially with high levels of uric acid. In addition, tasquinimod, one of the potential drugs identified in our study, was shown to ameliorate the pulmonary vascular remodeling in Sugen 5416/hypoxia-induced PH rat models *via* the inhibition of the class IIA subset of histone deacetylases and further increase of myocyte enhancer factor 2, which is crucial for maintaining pulmonary vascular homeostasis and RV function (Sofer et al., 2018). However, it should be cautious for the repurposing of the 27 potential drugs before more studies are carried out to give more information on their impacts on PH pathogenesis.

The present study demonstrated a reduction in 10 hub genes including *NCF2*, *MNDA/Ifi211*, *HCK*, *FGR*, *CSF3R*, *AQP9*, *S100A8*, *G6PD/G6pdx*, *PGD*, and *TXNRD1* in lungs of severe PH patients of the GSE24988 dataset as well as in lungs of hypoxic PH mice. Among these genes, *G6PD* has been directly correlated with PH. A recent study showed that *G6PD* deficiency caused metabolic abnormality, especially an upregulation of oxidative stress, thus facilitating spontaneous PH in mice (Varghese et al., 2021). A reduction of G6PD expression was found in rat lung tissues at an early stage of PH induced by one-dose Sugen 5416 injection and subsequent hypoxia challenge for two weeks (Varghese et al., 2020). However, the role of *G6PD* in PH pathogenesis is controversial. Another study showed that hypoxia-induced PH was prevented by *G6PD* deficiency, and that established severe PH in *Cyp2c44* ^−/−^ mice was ameliorated by the knockdown with *G6PD* shRNA or by G6PD inhibition (Joshi et al., 2020). Therefore, the divergency of the effect of *G6PD* on pulmonary vascular remodeling might lie in the different stage of the disease under investigation and the pathological stress giving rise to PH. More studies are warranted to confirm the role of *G6PD* in the disease setting. In line with our findings, the study by Neubert et al. (2020) showed a decreased expression of FGR in pulmonary veno-occlusive disease (PAH with overt features of venous/capillary involvement) lung explants compared to that of healthy control samples. Li et al. (2020) conducted a comprehensive miRNA–mRNA network analysis and identified that *AQP9* was one of the five hub genes possibly involved in the pathogenesis of idiopathic PAH. *TXNRD1* was also decreased and identified as an iron metabolism-associated hub gene in lungs of idiopathic PAH (Zou et al., 2021). *S100A8*, a ligand of receptor for advanced glycation end products, was overexpressed in PAH patient-derived pulmonary artery smooth muscle cells compared to those from non-PAH control subjects in the absence of any external growth stimulus (Nakamura et al., 2018). Although it is inconsistent with the reduction of *S100A8* in our study, one of the possible reasons would be the cellular heterogeneity in lung tissues, since they only examined its expression in pulmonary artery smooth muscle cells. Taken together, this detailed molecular mechanism relevant to these hub TRGs should be addressed elaborately in the future.

Some limitations should also be noted in this study. First, it is difficult to discern the causality between TRGs and Treg abundance due to the cross-sectional design of the original datasets. Second, we had no access to the clinical measures of the patients of the datasets. Therefore, the ROC curve for the distinction of PH patients from controls could not be adjusted by hemodynamic indicators or other traditional risk factors.

## Conclusion

We identified TRGs by virtue of the transcriptomic data of lung tissues and Treg infiltrates in datasets of PAH patients followed by the validation in experimental PH mice. This would shed some light on the Treg-associated therapeutic targets in the progression of PH and emphasize on a multigene signature as a novel indicator for PH.

## Abbreviations

AUC, area under the curve; DAVID, Database for Annotation, Visualization, and Integrated Discovery; DEGs, differentially expressed genes; FC, fold change; GEO, Gene Expression Omnibus; HIF-1α, hypoxia-inducible factor-1α; LV, left ventricle; PAH, pulmonary arterial hypertension; PH, pulmonary hypertension; PPI, protein–protein interaction; ROC, receiver operating characteristic; RT-PCR, real time polymerase chain reaction; RV, right ventricle; RVSP, right ventricular systolic pressure; SEM, standard error of the mean; Tregs, regulatory T cells; TRGs, regulatory T cell-related genes.

## Data Availability

The datasets presented in this study can be found in online repositories. The names of the repository/repositories and accession number(s) can be found in the article/Supplementary Material.

## Abbreviations

AUC, area under the curve; DAVID, Database for Annotation, Visualization, and Integrated Discovery; DEGs, differentially expressed genes; FC, fold change; GEO, Gene Expression Omnibus; HIF-1α, hypoxia-inducible factor-1α; LV, left ventricle; PAH, pulmonary arterial hypertension; PH, pulmonary hypertension; PPI, protein‐protein interaction; ROC, receiver operating characteristic; RT-PCR, real time polymerase chain reaction; RV, right ventricle; RVSP, right ventricular systolic pressure; SEM, standard error of the mean; Tregs, regulatory T cells; TRGs, regulatory T cell-related genes.
