# Supplementary material for: Regulatory T Cell-Related Gene Indicators in Pulmonary Hypertension
Source: Front Pharmacol. 2022 May 31;13:908783. doi: 10.3389/fphar.2022.908783 (PMC9197497; doi:10.3389/fphar.2022.908783)
Supplement: Supplementary file 1 [file DataSheet1.pdf]

Supplementary Materials

Regulatory T cell-related gene indicators in pulmonary hypertension

Yan Liu[1,*], Jun-Zhuo Shi[2,3], Rong Jiang[4], Shao-Fei Liu[5,6], Yang-Yang He[2,3], Emiel P.C. van der Vorst[7-11], Christian Weber[7,8,12,13], Yvonne Döring[7,8,14], Yi Yan[7,*]

1. Department of Nuclear Medicine, The First Affiliated Hospital of Zhengzhou University, Zhengzhou, China
2. School of Pharmacy, Henan University, Henan, China
3. College of Traditional Chinese Medicine, Henan University, Henan, China
4. Department of Cardio-pulmonary Circulation, Shanghai Pulmonary Hospital, Tongji University, Shanghai, China
5. Institute of Physiology, Charité - Universitätsmedizin Berlin, Corporate Member of Freie Universität Berlin, Humboldt-Universität zu Berlin, and Berlin Institute of Health, Berlin, Germany
6. DZHK (German Centre for Cardiovascular Research), Partner Site Berlin, Berlin, Germany
7. Institute for Cardiovascular Prevention, Ludwig-Maximilians-University Munich, Munich, Germany
8. DZHK (German Centre for Cardiovascular Research), Partner Site Munich Heart Alliance, Munich, Germany
9. Interdisciplinary Center for Clinical Research (IZKF), RWTH Aachen University, Aachen, Germany
10. Institute for Molecular Cardiovascular Research (IMCAR), RWTH Aachen University, Aachen, Germany
11. Department of Pathology, Cardiovascular Research Institute Maastricht (CARIM), Maastricht University Medical Centre, Maastricht, Netherlands
12. Department of Biochemistry, Cardiovascular Research Institute Maastricht (CARIM), Maastricht University Medical Centre, Maastricht, Netherlands
13. Munich Cluster for Systems Neurology (SyNergy), Munich, Germany
14. Department of Angiology, Swiss Cardiovascular Center, Inselspital, Bern University Hospital, University of Bern, Switzerland

*__Correspondence:__

Yi Yan, Institute for Cardiovascular Prevention, Ludwig-Maximilians-University Munich, Pettenkoferstreet 8a, 80336 Munich, Germany. E-mail: yannie0928@163.com

Yan Liu, Department of Nuclear Medicine, The First Affiliated Hospital of Zhengzhou University, No. 1 Jianshe East Road, 450052 Zhengzhou, Henan Province, China. E-mail: lyly.smile@qq.com

Supplementary Table 1. Selective potential drugs targeting 10 hub TRGs

Drug	Gene	Interaction Type	Sources	Query Score	Interaction Score
Pegfilgrastim	CSF3R	agonist	TdgClinicalTrial, ChemblInteractions, TEND, GuideToPharmacology, TTD	48.15	100.02
Balugrastim	CSF3R	agonist	ChemblInteractions	4.38	9.09
Lipegfilgrastim	CSF3R	agonist	ChemblInteractions	4.38	9.09
PLX-5622	CSF3R	n/a	TTD	4.38	9.09
Reparixin	CXCR1	modulator	ChemblInteractions, GuideToPharmacology, TTD	6.57	19.1
Navarixin	CXCR1	antagonist	TdgClinicalTrial, GuideToPharmacology, TTD	4.38	12.73
Rasburicase	G6PD	n/a	PharmGKB, FDA	24.51	5.02
Phenazopyridine	G6PD	n/a	PharmGKB	21.88	4.48
Pegloticase	G6PD	n/a	PharmGKB, FDA	17.51	3.59
Mafenide	G6PD	n/a	PharmGKB, FDA	13.13	2.69
Co-trimoxazole	G6PD	n/a	NCI	8.75	1.79
Sulfanilamide	G6PD	n/a	PharmGKB	8.75	1.79
Chlorproguanil	G6PD	n/a	PharmGKB	8.75	1.79
Succimer	G6PD	n/a	PharmGKB, FDA	8.75	1.79
Sulfadiazine	G6PD	n/a	PharmGKB, FDA	6.57	1.34
Carmustine	GSR	inhibitor	TdgClinicalTrial, ChemblInteractions, TEND	8.75	4.24
Oxiglutatione	GSR	inhibitor, substrate	ChemblInteractions, GuideToPharmacology	6.57	6.37
Alanyl glutamine	GSR	n/a	TTD	4.38	4.24
CP-461	PDE2A	n/a	TTD	4.38	12.73
BPN-14770	PDE4D	modulator	GuideToPharmacology, TTD	8.75	4.24
Dyphylline	PDE4D/ PDE7B	inhibitor	TdgClinicalTrial, TEND	7.5/ 5	1.82/ 9.09
Difamilast	PDE4D	inhibitor	GuideToPharmacology	4.38	2.12
Piclamilast	PDE4D	n/a	TTD	4.38	2.12
Atogepant	S100A12	n/a	TTD	4.38	12.73
Tasquinimod	S100A9	n/a	TTD	4.38	31.83
Paquinimod	S100A9	n/a	TTD	4.38	31.83
Fotemustine	TXNRD1	n/a	TTD	6.57	31.83

n/a, not available; TEND, Trends in the exploitation of novel drug targets; TTD, Therapeutic Target Database; FDA, Food and Drug Administration.

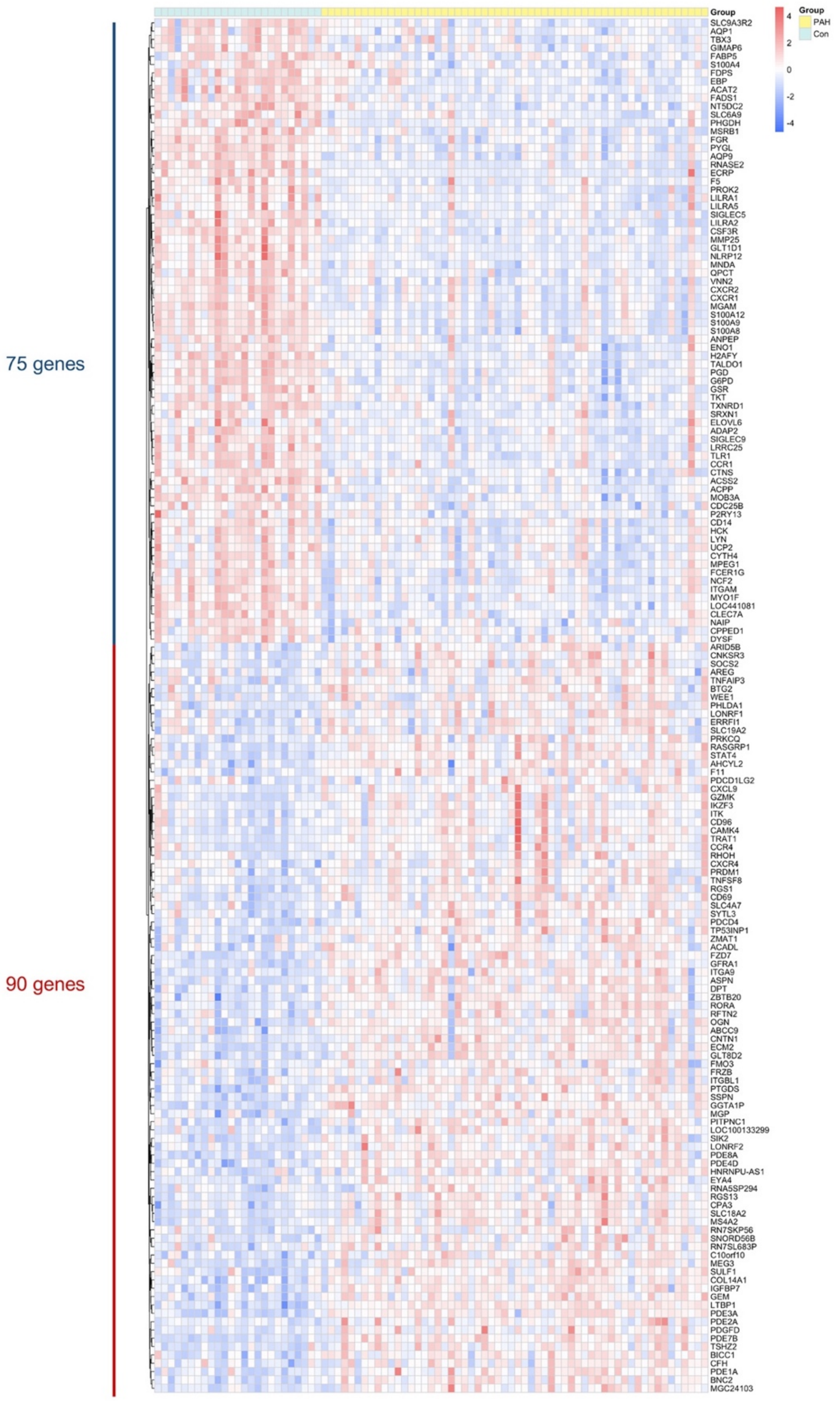

Supplementary Figure 1. Tregs related genes (TRGs) in each lung sample of PAH patients and controls. The expression of 165 TRGs (90 upregulated and 75 downregulated in PAH vs. controls) in each lung sample of dataset GSE117261 were displayed in heatmap. The yellow cell stands for lung sample of PAH patient and the light green for that of controls.

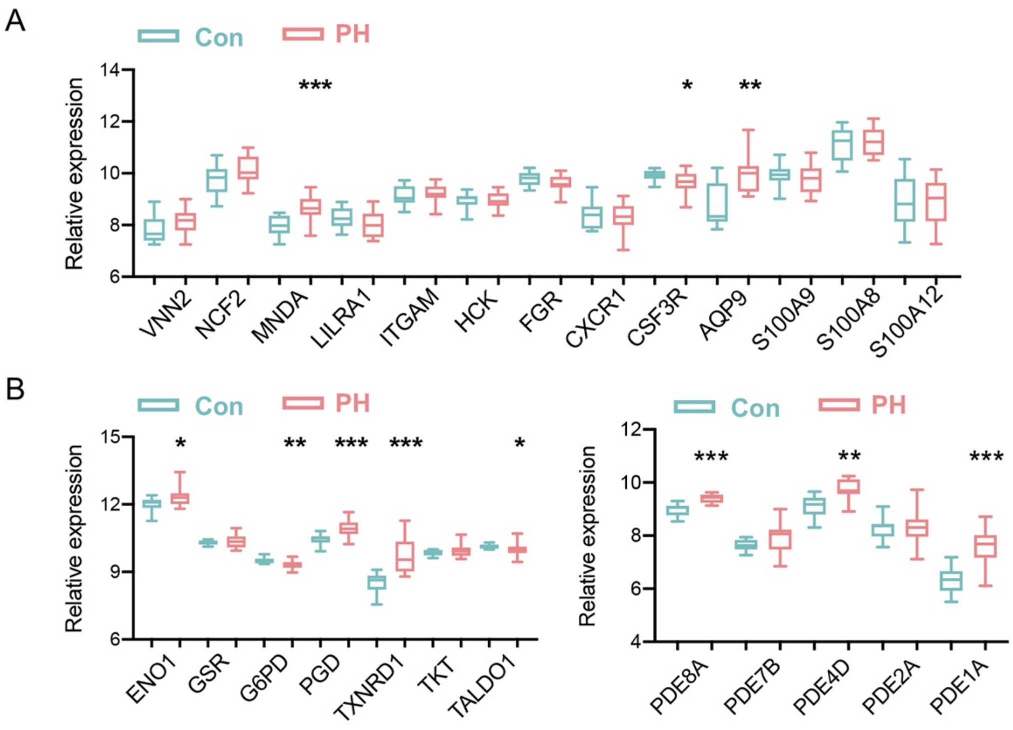

Supplementary Figure 2. Validation of hub TRGs in lungs of PH patients.
(A-C) The gene expression of hub TRGs in first (left) cluster (A), hub TRGs in second (middle) cluster (B) and hub TRGs in third (right) cluster (C) of Figure 3B were examined in lungs of 15 PH patients and 11 controls of dataset GSE113439. Data represent mean ± SEM. * $P < 0.05$; ** $P < 0.01$; *** $P < 0.001$ compared to control subjects, as analyzed by unpaired *t* test or Mann-Whitney test as appropriate.
